# Omics Integration Analyses Reveal the Early Evolution of Malignancy in Breast Cancer

**DOI:** 10.3390/cancers12061460

**Published:** 2020-06-04

**Authors:** Shamim Sarhadi, Ali Salehzadeh-Yazdi, Mehdi Damaghi, Nosratollah Zarghami, Olaf Wolkenhauer, Hedayatollah Hosseini

**Affiliations:** 1 Stem Cell Research Center, Tabriz University of Medical Sciences, Tabriz 5166614756, Iran; shamim.sarhadi65@gmail.com (S.S.); zarghami@tbzmed.ac.ir (N.Z.); 2 Department of Medical Biotechnology, Faculty of Advanced Medical Sciences, Tabriz University of Medical Sciences, Tabriz 5166614756, Iran; 3 Department of Systems Biology and Bioinformatics, University of Rostock, 18051 Rostock, Germany; ali.salehzadeh-yazdi@uni-rostock.de (A.S.-Y.); olaf.wolkenhauer@uni-rostock.de (O.W.); 4 Department of Oncologic Sciences, Morsani College of Medicine, University of South Florida, Tempa, FL 33612, USA; Mehdi.Damaghi@moffitt.org; 5 Department of Cancer Physiology, Moffitt Cancer Center and Research Institute, Tampa, FL 33612, USA; 6 Experimental Medicine and Therapy Research, University of Regensburg, 93053 Regensburg, Germany

**Keywords:** breast cancer, cancer evolution, omics data integration, machine learning, forward and backward evolution

## Abstract

The majority of cancer evolution studies involve individual-based approaches that neglect the population dynamics necessary to build a global picture of cancer evolution for each cancer type. Here, we conducted a population-based study in breast cancer to understand the timing of malignancy evolution and its correlation to the genetic evolution of pathological stages. In an omics integrative approach, we integrated gene expression and genomic aberration data for pre-invasive (ductal carcinoma in situ; DCIS, early-stage) and post-invasive (invasive ductal carcinoma; IDC, late-stage) samples and investigated the evolutionary role of further genetic changes in later stages compared to the early ones. We found that single gene alterations (SGAs) and copy-number alterations (CNAs) work together in forward and backward evolution manners to fine-tune the signaling pathways operating in tumors. Analyses of the integrated point mutation and gene expression data showed that (i) our proposed fine-tuning concept is also applicable to metastasis, and (ii) metastases sometimes diverge from the primary tumor at the DCIS stage. Our results indicated that the malignant potency of breast tumors is constant over the pre- and post-invasive pathological stages. Indeed, further genetic alterations in later stages do not establish de novo malignancy routes; however, they serve to fine-tune antecedent signaling pathways.

## 1. Introduction

Modern cancer biology, which is expanding rapidly in the light of omics approaches, has validated many aspects of Darwinian somatic evolution in cancer progression [1]. Such evolutionary logic influences our thinking and actions [2]. However, there are still some unknowns in the cancer evolution field, and innovative ideas and approaches are needed. For example, it is not clear how the progress of tumor staging could be correlated to clinical outcomes. It is speculated that tumor staging is the leading prognosis indicator and that the decline in cancer mortality in diverse cancer types is predominantly the result of screening, which reduces the incidence of larger tumors [3,4,5,6,7,8]. However, it has been suggested that certain features of tumors have more prognostic relevance than the anatomy of the tumor [9]. Furthermore, recent studies in breast and colorectal cancer have shown that disseminated cancer cells from earlier stages are the main cause of metastases [10,11]. Hence, it is still unclear to what extent the evolution of malignancy represents the evolution of genetic alterations and pathological progression of tumors.

The study of the somatic evolution of cancer relies on the dynamic genotype‒phenotype relationship of cancer cells at different stages of tumor progression [12]. Data for cancer evolutionary studies are mostly obtained via paired or longitudinal sampling from individual patients [13,14]. However, this approach is technically complicated and has some limitations by default. For example, multiple sampling and biopsies are not adequate to cover the whole heterogeneous tumor population [14,15]. Moreover, the depth of sequencing that is used to generate the mutational portrait of samples is still below the detection limit for minor subclones within the samples [16]. Most importantly, genetic drift, genetic background, and neutral evolution introduce undetectable bias into individual-based evolutionary studies [17,18]. Recently, the Pan-Cancer Analysis attempted to draw maps of the similarities and differences among the genomic and cellular alterations in a population across diverse tumor types, reflecting some evolutionary traces [19]. Nonetheless, there is still a gap in the exploration of cancer evolution within the population of each cancer type.

Studying the evolution of malignancy in large populations of patients will solve some of the mentioned issues, and might allow new aspects of cancer evolution that are not visible in the individual-based approaches to be pinpointed. We exploited a large volume of available data on different stages of breast cancer to build a universal portrait of tumor evolution in breast cancer. The most common type of breast cancer is ductal carcinoma, which consists of two main stages, ductal carcinoma in situ (DCIS) and invasive ductal carcinoma (IDC) [20]. DCIS or Stage 0 breast cancer is considered the noninvasive stage, and is the immediate precursor of the most invasive breast cancers [20,21].

With the advent of high-throughput technologies, researchers can quantify cellular changes at different molecular levels. Omics data have been broadly used for disease classification and identification of molecular gene signatures, but low reproducibility is a major weakness of these approaches [22]. However, new approaches based on omics data integration are expected to play a key role in identifying and qualifying new biomarkers, which is why the focus of data analysis approaches has shifted from single-omics to multi-omics data integration [23,24]. Consequently, many integrative methods, tools, and packages have been devised to identify cancer driver genes [25].

In this study, we used an omics integration approach to integrate the gene expression, copy-number alteration (CNA), and mutation data for the DCIS, IDC, and metastasis (Met) samples. Our results indicated that the malignant phenotype mostly forms in the earlier stages of tumor progression. Phylogenetic analyses depicted a global picture of breast cancer evolution that indicated early divergence of metastases at the DCIS stage. We discovered that further genetic alterations in advanced tumors do not generate new functional pathways in the tumor, but rather serve as fine-tuning effectors for antecedent changes from earlier stages. The fine-tuning happens via two different pathways, forward and backward evolution. In forward evolution, cancer cells increase the single gene alteration (SGA) burden to increase the efficiency of selected pathways. In backward evolution, tumor cells exploit CNAs in order to reverse the expression direction of unwanted inherited genetic changes, with a negative impact on the function of operating pathways. Therefore, we concluded that the malignancy fate is determined at the very early stages, and subsequent genetic changes are the fine-tuners of the evolutionary path.

## 2. Results

### 2.1. DCIS and IDC Are Indistinguishable Based on Gene Expression Profiles

In breast cancer progression, it is widely believed that DCIS and IDC are the noninvasive and invasive states of breast tumors, respectively [20,21]. We hypothesized that DCIS can be distinguished from IDC based on molecular features. Hence, we assessed the differences between normal, DCIS, and IDC samples using gene expression analyses. Accordingly, we used 10 datasets including 430 DCIS, IDC, and normal samples, profiled by three different platforms (Figure S1a). We used microarray data due to the availability of sufficient gene expression data for DCIS and IDC samples with a normal matched dataset. We then performed a statistical analysis for the identification of differentially expressed genes (DEGs), which resulted in 4275, 5000, and 1098 DEGs with a combined *p*-value < 0.05 for DEG1 (DCIS vs. normal), DEG2 (IDC vs. normal), and DEG3 (DCIS vs. IDC), respectively (Table S1 and Figure S1b). In order to guarantee that our results were not biased because of the faults of microarray technology, we compared DEG2 with an analogous DEG dataset from the largest available RNA-sequencing dataset in breast cancer [26] and found significant similarities between these two data groups (*p* < 0.0001, exact hypergeometric probability test; Figure S1c).

**Figure 1:**
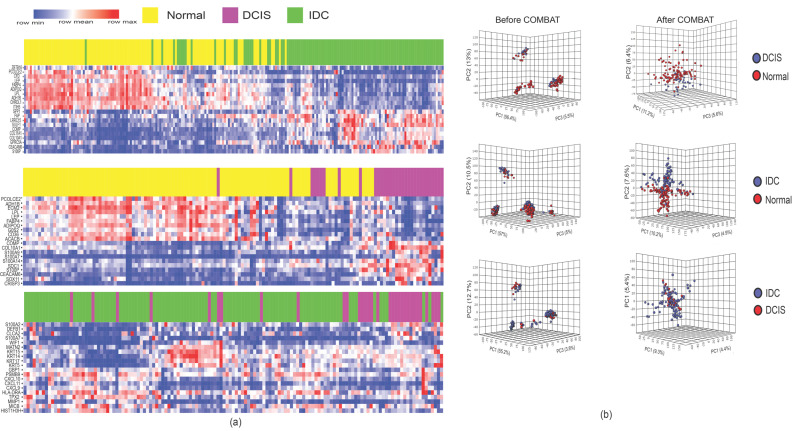
Unsupervised hierarchical clustering and PCA. (**a**) Two-dimensional heatmaps show 16 differentially expressed genes based on the log fold-changes. Clustering was done after applying the ComBat method; (**b**) PCA analyses before (left) and after ComBat (right). PCA distinguished DCIS and IDC from normal samples based on their gene expression profiles, but failed to distinguish DCIS from IDC samples.

After the identification of DEGs, we used principal component analysis (PCA) and hierarchical clustering to determine whether the gene expression profiles of DCIS and IDC samples were separable. We did not find a clear separation between these samples (Figure 1a,b). To avoid the bias of incompatibility noise in our analyses, we used Combining Batches (ComBat) method in the PCA and hierarchical clustering methods to remove the batch noise. Applying PCA and the hierarchical clustering method after ComBat was able to differentiate DCIS and IDC samples from normal samples. However, DCIS and IDC samples could not be differentiated even after applying ComBat (Figure 1a,b). These results indicated that DCIS is not distinguishable from IDC using gene expression clustering approaches.

### 2.2. Stage-Dependent Gene Expression Analyses Did Not Generate a Malignancy-Predictive Feature

**Figure 2:**
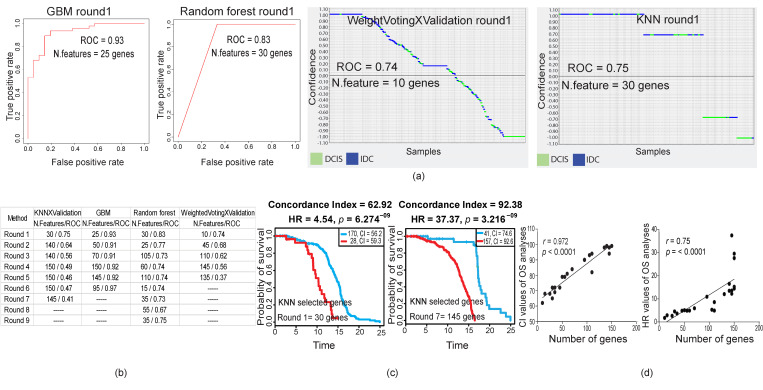
Machine-learning (ML) predictive models and survival analyses. (**a**) ROC plots and plots obtained by a prediction results viewer for the first round of feature selection by four different ML models. Separation of DCIS and IDC samples was achieved by both models. (**b**) ROC results of the first nine rounds of feature selection. After each round of feature selection, we excluded this gene list feature from the DEG source and repeated it for a new feature selection. Note that we did not continue feature generation if ROC/AUC was constant or dropped below 0.5. (**c**) Kaplan‒Meier survival analyses of the first and the last rounds of gene feature selection optimized using the KNN model. Note that the last feature with ROC below 0.5 (panel b) also strongly correlated with survival. All log rank tests were significant (*p* < 0.05; *p*-value generated by SurvExpress online server). (**d**) Correlation of concordance index (CI; left) and hazard ratio (HR; right) obtained from overall survival (OS) analyses with the number of genes in each gene feature (see Figure S2b; Pearson correlation test).

Here, we applied further advanced approaches to distinguish DCIS and IDC based on their gene expression profiles. Therefore, we used machine-learning (ML) methods to define a minimal number of genes as representatives of DCIS and IDC. We optimized four different ML methods (weightedVotingXValidation, KNN, GBM, and Random Forest) and they successfully generated distinctive (Receiver Operating Characteristics/Area Under the Curve, ROC/AUC > 0.5) features able to sort DCIS from IDC samples (Figure 2a). In order to evaluate the accuracy of selected gene features, we removed all of the first selected gene features and reran all ML models to generate new distinctive features. Notably, all ML methods were able to reconstruct new distinctive features, even after excluding previous selected gene features from the data source (Figure 2b and Figure S2a). Next, we hypothesized that the first selected gene features might have a stronger biological correlation to the clinical outcomes (e.g., survival) in patients as compared to the gene features in the next rounds (Figure 2c and Figure S2b). Our analyses revealed that the discriminative power for the selected features of our chosen ML methods did not indicate their biological relevance. Indeed, features with nonsignificant discriminative power (ROC/AUC < 0.5) were also strongly correlated to biological outcomes (Figure 2c and Figure S2b). Inspecting the survival analyses and selected features using ML methods, we realized that the strength of the hazard ratio (HR) and concordance index (CI) was strongly correlated to the number of genes in the selected features (*p* < 0.0001, Pearson correlation test; Figure 2d). Furthermore, we could not find significant consistency between the direction of expressed genes in the source of selected feature gene (DCIS or IDC) and the poor- and good-prognosis samples in the survival analyses (Figure S2c). These results suggested that the distinguishing power of the discriminative gene features of DCIS versus IDC was not correlated to the clinical outcomes and malignancy of breast tumors.

### 2.3. Stage-Dependent CNA and SGA Integrative Analyses Did Not Generate Malignancy-Predictive Features

**Figure 3:**
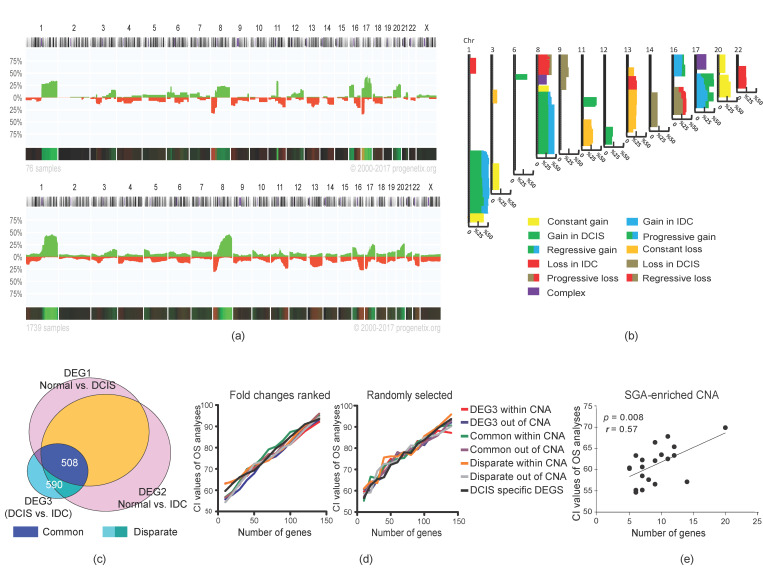
Integration of copy-number alteration (CNA) and single gene alteration (SGA) of DCIS and IDC. (**a**) CNA map of DCIS (up) and IDC (down) obtained from Progenetix database (DCIS = 76, IDC = 1739). The y-axis depicts the percentage of samples with aberrations (green stands for gain and red stands for loss) for each chromosomal region. (**b**) Map of CNA prototypes (see also Table S3). (**c**) A Venn diagram of common and disparate genes. (**d**) Correlation of concordance index (CI) obtained from overall survival (OS) analyses with the number of genes that selected based on ranked (left) or random (right) fold-changes to overall survival. Genes were selected from common, disparate, DCIS-specific, and DEG3 (IDC vs. DCIS) within and outside of CNAs. (**e**) Correlation of CI obtained from OS analyses with the number of genes in SGA-enriched CNA. The *p*-value in (**e**) was calculated by a Pearson correlation test.

The aggravation of chromosomal aberration is a general aspect of tumor progression in the evolutionary path toward advanced stages [27]. We speculated that the integration of CNA (copy-number alteration) and SGA (single gene alteration, extracted from DEGs) would help us to find a better discriminative feature for sorting DCIS from IDC samples. Indeed, we assumed that the integration of SGAs and CNAs would generate better discriminative features to distinguish DCIS and IDC in correlation with malignancy outcomes (e.g., survival analyses). To test this hypothesis, we extracted the CNA map of DCIS and IDC samples (Progenetix, database; version 2017; 76 DCIS and 1736 IDC samples; Figure 3a, and Table S2). We assumed that all cases of IDC had experienced a DCIS stage during tumor progression. Thus, we generated a combined map of the CNAs of DCIS and IDC to depict an evolutionary view of the CNA map for breast tumors (Figure 3b). This map generated 25 prototypes, including five prototypes that were specific for DCIS and three prototypes that were specific for IDC. There were two complex prototypes without clear gain and loss patterns in correlation with the DCIS or IDC stage (see Section 4.4 and Tables S2 and S3). We also found 15 shared prototypes between DCIS and IDC. On the other hand, not all SGAs were generated de novo in IDC. Almost half of the SGAs (508 out of 1098 for DEG3, extracted from IDC vs. DCIS) originated from DCIS (referred to herein as “common genes”), and their changes became stronger in IDC. The rest of the SGAs, which we refer to as “disparate genes”, including SGAs, were generated exclusively in the IDC stage (Figure 3c, Figure S3a, and Table S1). Next, we distributed the SGAs in CNAs and ranked them based on log fold-changes (LFC). We then tested if any of these gene sets (common/disparate, within/not within CNAs) had a better CI to the survival outcomes. For this analysis, we selected features of 10 genes (arbitrary number) and added 10 more genes for the next features. We performed these analyses for the fold-changed ranked gene lists and for the random list. Interestingly, the CIs of all groups of SGAs were correlated to the number of genes in each feature and not to their CNA-related category (Figure 3d and Figure S3b). We further assessed whether SGA-enriched parts of chromosomal regions within CNAs (regions with many SGAs, with not more than 5 Mb distance to the next SGA; Table S3) might have a stronger correlation to malignancy outcomes compared to other chromosomal regions. Our results recapitulated previous findings that the number of genes defines the strength of CI, and not the specific region (*p* = 0.008, Pearson correlation test; Figure 3e). Furthermore, we could not find a significant consistency between the directions of expressed genes in the source of selected feature genes (IDC) and the poor- and good-prognosis samples in the survival analyses (Figure S3c). Finally, we tested several well-known gene signatures that are used in clinical settings and found that the number of genes in these signatures strongly correlated to their CI power (Figure S3d). Altogether, these results suggest that the increase in genetic alterations in IDC as compared to DCIS does not necessarily increase the malignant properties of tumors.

### 2.4. Genetic Changes in Earlier Tumor Stages Were More Functional Than in Later Stages

**Figure 4:**
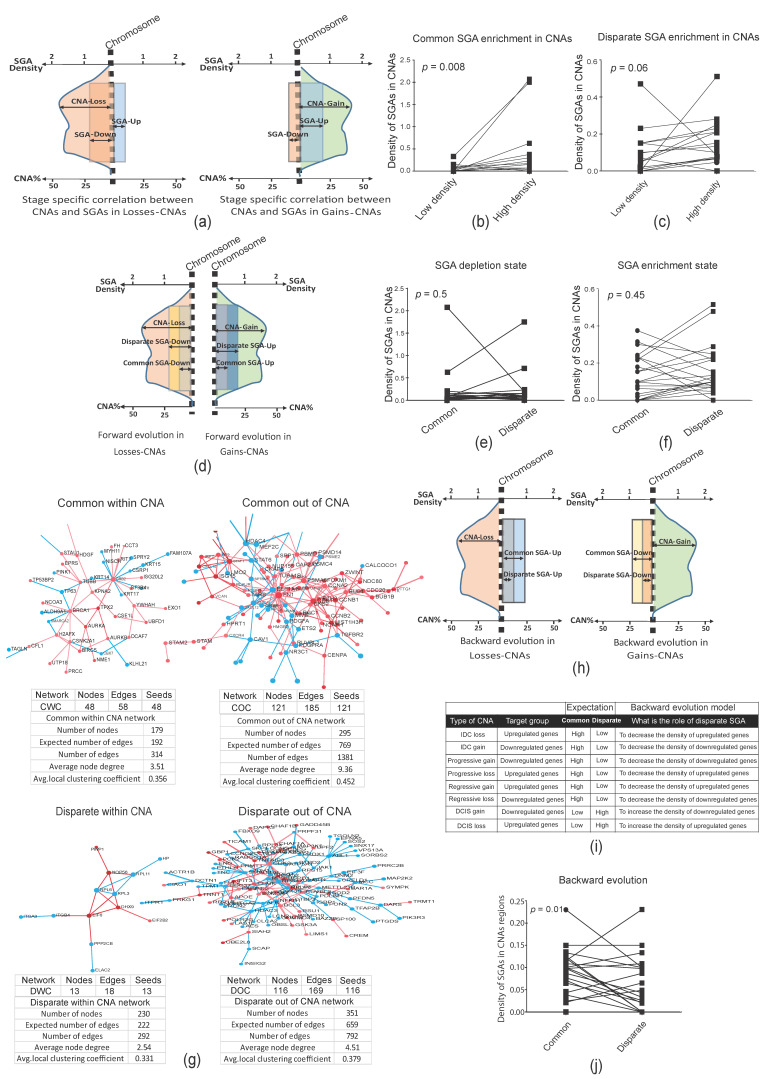
Evolutionary correlation models of SGAs and CNAs. (**a**) This scheme depicts the expectation of stage-specific correlation between the direction of CNAs and SGAs. It was expected that the density of upregulated SGAs would be higher than that of downregulated SGAs in gain CNAs (right) and the density of downregulated SGAs would be higher than that of upregulated SGAs in loss CNAs (left). (**b**) Common SGAs were correlated with the direction of CNA prototypes of DCIS. The density of downregulated genes was high in loss CNAs and the density of upregulated genes was high in gain CNAs (see Figure S4a). (**c**) Disparate SGAs were correlated with the direction of CNA prototypes of IDC. The density of downregulated genes was high in loss CNAs and the density of upregulated genes is high in gain CNAs (see also Figure S4b). (**d**) This scheme depicts the expectation of forward evolution for the common and disparate SGAs in IDC CNAs. It was expected that the density of disparate upregulated SGAs would be higher than that of common upregulated SGAs in gain CNAs (right), and that the density of disparate downregulated SGAs would be higher than common downregulated SGAs in loss CNAs (left; see also Figure S4c). (**e**,**f**) The forward evolution model was not confirmed either for the SGA-depletion state (**e**) or the SGA-enrichment state (**f**; see Figure 4c). (**g**) Specific protein‒protein interaction networks (PPINs) of common SGAs within CNAs (CWC), common outside of CNAs (COC), disparate within CNAs (DWC), and disparate outside of CNAs (DOC). Networks were constructed by mapping gene sets to the human PPIN database. Blue and red nodes represent down- and upregulated genes, respectively. PPINs statistics were calculated by STRING (https://string-db.org/). (**h**) This scheme depicts the expectation of backward evolution for the common and disparate SGAs in IDC CNAs. It was expected that backward evolution would decrease the density of upregulated SGAs in loss CNAs (left) and the density of downregulated SGAs in gain CNAs (right). (**i**) The table presents a backward evolution concept (the numbers for this prediction are presented in Figure S4e). Note that we evaluated all SGAs (common and disparate) in the IDC CNAs or progressive CNAs prototypes as the final evolved version of CNAs. (**j**) Evaluation of backward evolution. *p*-values in panels (**b**,**c**,**e**,**f**,**j**) were calculated by Wilcoxon matched-pair signed rank test.

The constant malignant properties of tumors in premalignant stages raise a question about the role of the accumulation of genetic alterations in later stages. To address this question, we first explored the functions of earlier and later events during tumor evolution, as well as the possible interactions between them. We assumed that each CNA prototype is an evolutionary unit (Figure 3b) wherein we can assess the correlation between the direction of CNA and SGA changes during the progression of tumors from DCIS to IDC. The IDC has two types of gene expression profile: common, which originates from DCIS; and disparate, which is acquired specifically in IDC (see Figure 3c and Table S1). In addition, there are different types of CNAs for IDC and DCIS: DCIS-specific, which only exists in DCIS; progressive CNAs, which are inherited from DCIS and progress further in IDC; IDC-specific, which is acquired specifically in IDC; and constant CNAs, which are constant changes (gain or loss of function) in DCIS and IDC. First, we calculated the density of common and disparate up- and downregulated genes in their CNA prototypes (SGA density is the number of SGA/Mb; Table S4). We then specifically tested whether the expression direction of common and disparate SGA density was similar to the direction of CNAs in each stage (Figure S4a). Our results revealed a positive correlation between the expression direction of SGAs and the direction (gain or loss) of CNAs in each tumor stage (*p* = 0.008 for common genes in constant CNAs and DCIS CNAs, *p* = 0.06 for disparate genes in constant CNAs and IDC CNAs, Wilcoxon matched-pair signed rank test; Figure 4a–c and Figure S4a,b). We then sought a serial relationship between common and disparate SGAs and their correlation to the direction of CNAs. Since the direction of changes of SGAs and CNAs in the stage-dependent analyses were harmonic and DCIS and IDC had indistinguishable gene expression profiles, we speculated a positive correlation between the direction of IDC CNAs and the direction of changes for both common and disparate SGAs. Here, we assumed a forward evolution model in which the disparate SGAs increased the density of SGAs in the direction (up- or downregulation) of existing common SGAs in the IDC-specific and IDC-progressive CNAs (Figure 4d and Figure S4c). Surprisingly, we could not confirm the forward evolution model for the consequential common and disparate SGAs in the CNAs related to IDC (Figure 4d–f). Subsequently, we analyzed the functional interactions of common and disparate SGAs using protein‒protein interaction networks (PPINs). We separated SGAs into two categories: SGAs within and outside of CNAs. Interestingly, we found that common SGAs were the most interactive in the specific PPIN analyses, as shown by the higher average node degree (Figure 4g). Notably, SGAs within CNAs were poorly connected in the networks. In addition, gene-set enrichment analyses (GSEA) revealed the enrichment of more functional pathways for the common gene sets compared to disparate genes and SGAs in the CNAs (Figure S4d).

Up to this point, our results suggested that the progression of CNAs in later stages did not increase the density of SGAs in each given CNA. Surprisingly, further genetic alterations specific to the later stages did not generate further signaling pathways in the tumor, contradicting a forward evolution model for extra genetic alterations in the later stages. Hence, we proposed two scenarios to explain the role of further genetic alterations in the later stages. The first scenario was to consider a neutral evolution model whereby further genetic alterations randomly accumulate in tumors. This scenario might be accurate, but is difficult to test. In a substitute scenario, we hypothesized a backward evolution in the later stages, serving to reverse the expression of unwanted genes inherited from earlier stages. In a backward evolutionary model, we supposed that CNAs in the later stages would function in the reverse direction to SGAs from earlier stages within that region (Figure 4h,i). For example, the forward evolution model supposed an additive impact whereby an IDC CNA gain increases the density of upregulated SGAs (which failed; see Figure 4d,f); however, the backward evolution model posited that an IDC CNA gain is selected to decrease the density of downregulated SGAs in that given chromosomal region, inherited from the DCIS stage (Figure 4i). Our results corroborated this hypothesis in that we found that later CNAs reversed the density of common SGAs in those regions (*p* = 0.01, Wilcoxon matched-pair signed rank test; Figure 4j and Figure S4e). This hypothesis was further supported by CNAs being constant (the percentage of gain or loss was constant) through the DCIS and IDC stages. In these constant CNAs, the density of common and disparate SGA did not change in the forward or backward models (Figure S4f–h). Therefore, we concluded that CNAs could serve as backward evolutionary tools in later stages to reverse the expression of some of the SGAs inherited from earlier stages.

### 2.5. Forward and Backward Evolution Models Were Exploited for the Fine-Tuning of Operating Pathways during Tumor Progression

To better understand the role of early and late genetic alterations during tumor progression, we performed more functional analyses. Since in the late stage of tumors, genetic alterations are the accumulative results of the early (common SGAs) and late stages (disparate SGAs), we studied the function of disparate SGAs as a part of the whole running program (common + disparate) in the late stage. Therefore, we performed PPIN analyses for common SGAs and all DEG3 (all common and disparate SGAs), independent of their chromosomal loci. Surprisingly, we found that the higher connectivity of the network suggested more intricate biological functions for the protein network of all DEG3 compared to the common SGAs (Figure S5a). We then performed GSEA for both networks and found that the common SGA profile was mostly related to cell proliferation pathways. Interestingly, all of these proliferation-related pathways were enriched in DEG3, which indicates the constant role of selected pathways during tumor progression (Figure S5b and Table S5).

**Figure 5:**
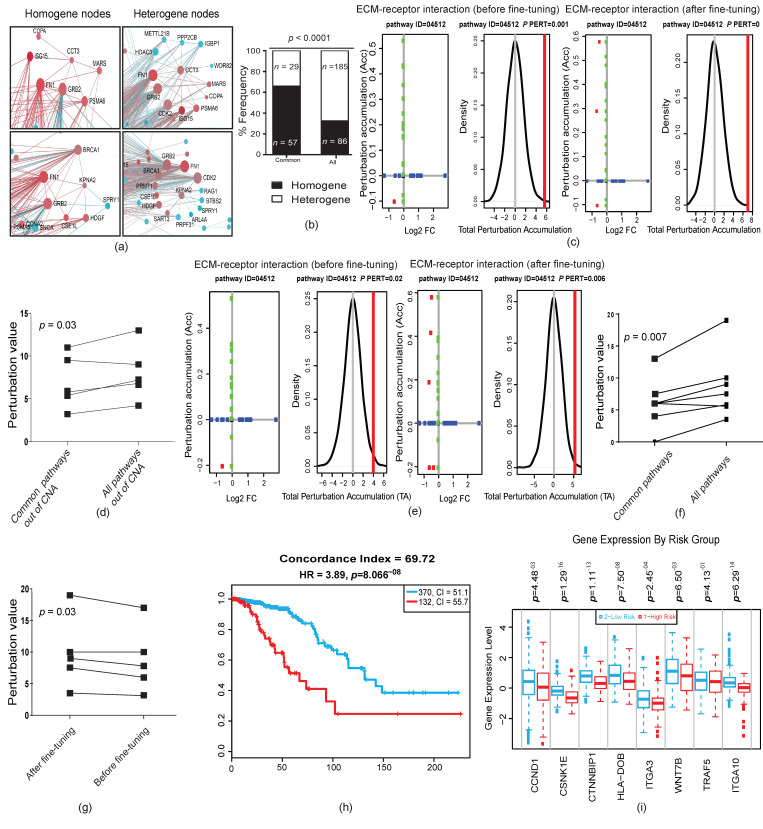
Forward and backward evolution and fine-tuning concept. (**a**) Examples of the direction of changes in homogene and heterogene nodes; (**b**) homogeneity and heterogeneity statistical analysis for common and all (all DEG3) networks; (**c**) examples of signaling pathway impact analyses (SPIA) for analyses of common SGAs and all DEG3 (common + disparate) outside of CNAs (see Table S7). In this plot, the horizontal axis represents the *p*-value (minus log of) corresponding to the probability of randomly obtaining at least the observed number of genes (NDE) on the given pathway. The vertical axis represents the *p*-value (minus log of) corresponding to the probability of randomly obtaining the observed total accumulation (tA) or more extreme on the given pathway. Unchanged genes were assigned 0 log2 fold-change. The null distribution of the net accumulated perturbations is also given (right panel). The observed net accumulation tA is shown as a red vertical line (see [28]). (**d**) Evaluation of perturbation between common SGAs and all DEG3-SGAs outside of CNAs. Note that the numbers represent the pure changes of perturbation compared to the Table S7, which shows negative perturbation values for the inhibition of pathways and positive perturbation values for the activation of pathways. (**e**) Examples of SPIA analyses of common SGAs and all DEG3; (**f**) evaluation of perturbation between common SGAs and all DEG3-SGAs; (**g**) evaluation of perturbation between enriched pathways for all DEG3-SGAs before adding the eight backward-evolution genes and after adding backward-evolution genes; (**h**,**i**) survival analyses for eight backward-evolution genes (**h**). The direction of backward-evolution genes was in the direction of the good-prognosis samples (**i**). Evaluation *p*-values in (**d**,**f**,**g**) were calculated using a paired Student’s *t*-Test, and in (**b**,**k**,**l**) using a chi-square test.

We then investigated how further genetic alterations might affect the function of selected pathways from earlier stages. Closer inspection of PPINs suggested that highly interactive nodes in the common PPINs were more homogenous in their connections compared to the PPINs of DEG3 (a similar color, which means similar direction of expression; Figure 5a and Figure S5c). Thus, we assessed the homogeneity and heterogeneity of highly interactive protein nodes in the PPINs. For this purpose, we analyzed all nodes with ≥4 connections and considered them to be homogeneous nodes if ≥75% of connections showed a similar direction of expression (up-/downregulation). Our analyses revealed that the homogeneity of highly interacting protein nodes in common genes that originated from DCIS stages was 2‒3 times greater than at later stages (*p* < 0.0001, chi-square test; Figure 5b, and Table S6). These results, along with the information from GSEAs (Figure S5b), suggested that earlier operating pathways stay constant during tumor progression; however, the relationships between the involved components become more complex.

To investigate the functionality of the acquired complexity, we expanded our analyses by using signaling pathway impact analyses (SPIA) to track the functions of changes in the enriched pathways. The biggest advantage of SPIA is its ability to quantify the direction of changes by integrating the effect of topological positioning, gene expression values, and the impact of players’ interaction (inhibition or activation) in a given enriched pathway [28]. Therefore, we performed SPIA for (i) common SGAs out of CNAs as an earlier event in comparison with all SGAs of DEG3 out of CNAs, and (ii) all common SGAs as compared to all DEG3 to understand the functional impact of SGAs within the CNAs in the enriched pathways. Our analyses for the SGAs (only SGAs out of CNAs) showed that only five pathways had significant perturbation for the common profile (Figure 5c, Table S7). Next, we found that the perturbation of enriched pathways for common SGAs increased when we tested all SGAs (only SGAs out of CNAs; Figure 5d; *p* = 0.03 Student’s paired *t*-Test), which indicated that SGAs increase the function of selected pathways in a forward evolution manner. Similarly, we performed SPIA for all SGAs of common genes compared to all DEG3 (all SGAs within and outside of CNAs). Interestingly, perturbation was detected for only seven pathways (Figure 5e). Consistently, the perturbation values increased in the same enriched pathway in all DEG3 compared to the common profile (Figure 5f; *p* = 0.007, Student’s paired *t*-Test). These results indicated that further genetic alterations in later stages do not establish new paths for the operating pathways of tumor cells, but rather fine-tune and increase the function of descendant pathways, which can be explained by the forward evolution model.

In the next step, we functionally assessed the existence of backward evolution during tumor progression. In the previous section, we showed that CNAs in later stages serve to decrease the load of SGAs inherited from earlier stages in those regions where their function was left unaddressed (see Section 2.4 and Figure 4j). Here, we hypothesized that the reverting of SGAs by CNAs in the later stages increases the function of selected pathways in later stages. In this order, we sorted all genes from DEG1 (DCIS vs. normal; those are prone to reversal by CNA in later stages) that existed in the seven enriched pathways with significant perturbation from the SPIA results of DEG3 (Figure 5f). We found eight DCIS-upregulated genes in the CNA regions (six out of the seven enriched pathways) with a supposed backward evolution function (their expression reversed by CNA in later stage; CNA regions are listed in Figure S4e) and added them to the DEG3 along with their original gene expression values from DCIS and ran SPIA. Remarkably, the perturbation of these six pathways significantly decreased upon adding the mentioned eight genes from DCIS (Figure 5g; *p* = 0.03, Student’s paired *t*-Test; Figure S5d). We further tested these eight genes for correlation with the survival of patients with invasive breast carcinoma (TCGA data deposited in Survexpress), which showed significant prognostic value for the discrimination of poor- and good-prognosis samples. Interestingly, all of these genes had similar (higher) expression to their DCIS source in the good-prognosis samples compared to poor-prognosis ones (Figure 5h,i).

Altogether, these results indicated that SGAs in advanced stages, which are inherited from earlier stages, play a prominent role in the construction of functional signaling pathways. Those SGAs that are acquired in later stages serve as fine-tuners for the functions of SGAs that are inherited from earlier stages. These results further suggest that CNAs have no driving role in the evolution of functional signaling pathways of tumors, but, rather, are tools exploited by tumors for the fine-tuning in a forward or backward evolution model.

### 2.6. Metastases Diverge from Primary Tumor at Early Stages

**Figure 6:**
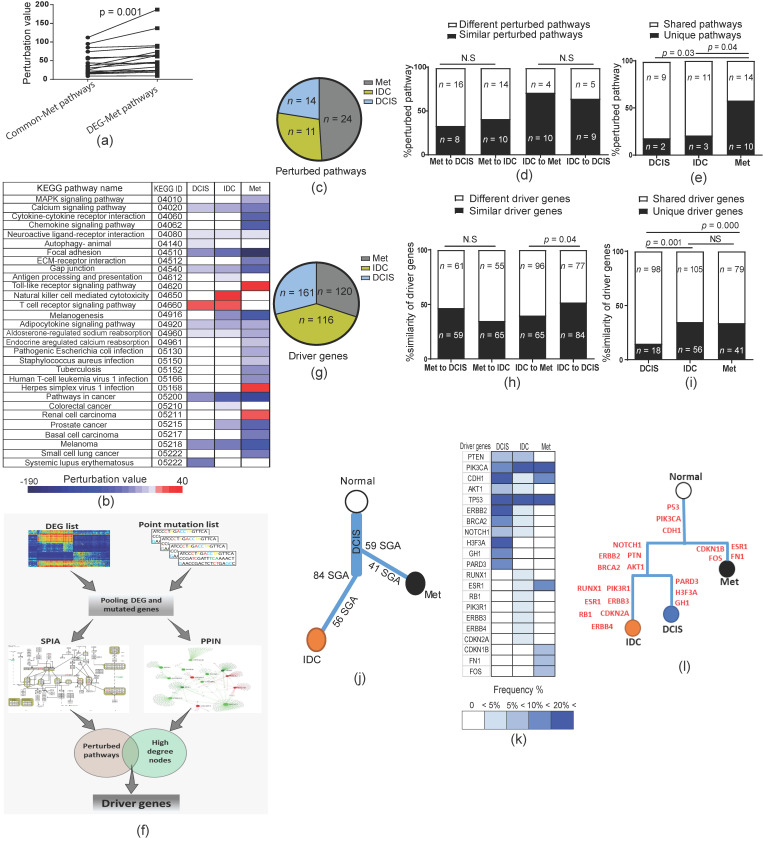
Evolution of metastasis and divergence time from primary tumor. (**a**) Evaluation of perturbation between common–Met SGAs and all DEG–Met SGAs; (**b**) heatmap of perturbed pathways obtained by SPIA analysis for DCIS, IDC, and Met; (**c**) pie chart presenting the number of perturbed pathways in each group; (**d**) similarity of perturbed pathways shown between the DCS, IDC, and Met groups (Table S9); (**e**) comparison of the number of unique and shared perturbed pathways for each group. A shared perturbed pathway is one that exists at least in two groups. (**f**) A demonstration of our modified version of DawnRank for the detection of driver genes (see Section 4.8); (**g**) a pie chart presenting the number of driver genes in each group; (**h**) the similarity of driver genes between the DCS, IDC, and Met groups; (**i**) comparison of the number of unique and shared driver genes for each group. A shared driver gene is the one that exists at least in two groups. (**j**) Conceptual phylogenetic tree extracted from (**d**,**e**,**h**,**i**); (**k**) heatmap of mutated driver genes shared between DCIS, IDC, and Met (see Table S10 and Figure S6b); (**l**) phylogenetic tree constructed based on the mutation profile of shared driver genes between DCIS, IDC, and Met, presented in (**k**). The valuation *p*-values in (**a**) were calculated by a paired Student’s *t*-test; those in (**d**,**e**) were calculated by Fisher’s exact probability test; and those in (**h**,**i**) were calculated by a chi-square test.

In cancer, metastasis is the demonstration of a malignant phenotypic evolution of primary tumors. Therefore, we tested the main aspects of our findings in metastatic (Mets) samples. For these analyses, we first generated a DEG for metastases as well as a common DEG list overlap between Met–DEG and DCIS–DEG profiles (common–Met, Figure S6a, and Table S8). We then ran the SPIA and generated pathways with significant perturbation. We identified 24 pathways for Met–DEG and 21 pathways for common–Met with significant perturbation values, whereas 19 of them were shared between the two analyses (Table S9). Consistent with our previous results for IDC (Figure 5c–f), fine-tuning was also detected between common–Met and Met–DEG pathways with significant perturbation (*p* = 0.001, Student’s paired *t*-test, Figure 6a). Furthermore, we ran SPIA for the DCIS–DEG list and compared perturbed pathways between DCIS, IDC, and Met. Interestingly, we found that metastasis-perturbed pathways were similarly shared between DCIS and IDC (Fisher’s exact probability test, Figure 6b–d). However, the number of perturbed pathways was significantly higher in metastasis samples (Fisher’s exact probability test, Figure 6b,e). Next, we ran a modified version of the DawnRank [29] analysis to identify the driver genes of each stage (Figure 6f, Table S10). Our analyses revealed that IDC had more shared driver genes with DCIS as compared to Mets and interestingly, the number of shared driver genes of Mets was similar between Met vs. DCIS and Met vs. IDC (chi-square test, Figure 6g,h, Figure S6b, Table S10). Furthermore, Met and IDC had significantly higher numbers of unique driver genes compared to DCIS (Figure 6i), implying a divergence from DCIS and further independent evolution. Our findings, which showed that (i) Met has more unique perturbed pathways compared to DCIS and IDC (Figure 6e), and (ii) Met has more unique driver genes compared to DCIS (Figure 6e), suggested that Met probably has more time for independent evolution as a result of neutral evolution in the absence of selection pressure [18,30]. Therefore, we hypothesized that phylogenetic analyses would show the early divergence of Met at the time of DCIS. Firstly, based on all similarities between perturbed pathways and driver genes (Figure 6d,e,h,i), we drew a conceptual phylogenetic tree that demonstrated the early divergence of metastasis at the DCIS stage (Figure 6j). We then drew an accurate phylogenetic tree using mutated genes in the shared driver gene profile between DCIS, IDC, and Met (Figure 6k), which consistently generated a similar picture to the conceptual phylogeny analyses (Figure 6l). Altogether, these results indicated that the evolution of a malignant phenotype in breast cancer is much faster than the evolution of pathological stages.

## 3. Discussion

As opposed to individual-based approaches, we conducted our evolutionary study on a population basis. This is because in individual-based approaches, (i) sampling methods do not cover the entire heterogeneity of tumors [14,15,16], (ii) individual sampling has the risk of deviation of interpretation due to genetic drift and neutral evolution [17,18], and (iii) the final candidates coming out of individual tumor evolution analyses can be biased by the environment or background of that given individual. In this study, we specifically aimed to address whether the evolution of the malignant phenotype of breast cancer represents the evolution of pathological stages. We selected ductal carcinoma, as it is the most frequent breast cancer type [20] and we had a reasonable number of samples with their matched adjacent normal tissue. Studying DCIS, IDC, and Met allowed us to generate an overall view of the stepwise Darwinian evolution of breast tumors.

Our results indicated that the different gene expression signatures obtained by different approaches may have discriminative power to separate DCIS from IDC samples; however, these gene expression signatures had nothing to do with the malignant properties of these samples. We further found that the increase in genomic alterations in later stages of tumor progression had no distinctive prognostic value. Our results indicated that SGAs, which are inherited from DCIS, play a prominent role in the construction of operating signaling pathways in later stages. Genetic alterations that appear in later stages play a fine-tuning role in terms of the function of genetic alterations inherited from earlier stages. Indeed, the majority of signaling pathways with significant perturbation in DCIS were also enriched in IDC and Met. Interestingly, cell motility pathways (e.g., Focal adhesion, Gap junction, and calcium signaling pathways) as the basis of cancer cell invasion were among the most perturbed pathways shared in DCIS, IDC, and Met. However, the same pathways showed three main differences in the advanced tumors as compared to their ancestral pathways from an early stage. First, the number of genes in the same pathways was higher in later stages compared to the ancestral ones, indicating the involvement of more genomic changes in the later stages. Second, the same pathways were increasingly heterogeneous in terms of the direction of the expression and regulation of their player in later stages. Third, the perturbation of enriched pathways significantly increased in the late stage, indicating fine-tuning evolution of the earlier-developed pathways by further genetic alteration in late-stage tumors.

We also found that the density of SGAs in the chromosomal regions with CNAs was higher compared to the regions without CNAs. In addition, the direction of CNAs turned out to be in the direction of SGAs in each stage, which was in line with the notion that CNA is one of the mechanisms of somatic gene deregulation [31]. We also found that the function of SGAs inside and outside of CNAs could be formulated in a forward evolution model with an additive impact on the fine-tuning of the signaling pathways of tumors. Surprisingly, we found that CNAs work partly in a backward evolution model, whereby this mechanism removes the negative regulators of selected signaling pathways during tumor progression. A recent punctuation evolution model speculated a driving role for CNAs, as it was found that the majority of CNAs are acquired at the earliest stages of tumor evolution, followed by stable clonal expansions forming the tumor mass [32]. Our analyses suggested that the evolution of tumors and especially the malignancy trait of tumors are mostly related to earlier SGA events outside of CNAs, as they were the main components of the perturbed pathways in IDC. In addition, the selected CNAs in later stages added no new functional pathways to the operating signaling pathways of advanced tumors as compared to DCIS. Moreover, the backward evolution mechanism that was detected in the CNAs hardly left room to interpret CNAs as the main driving force of tumor evolution, but rather indicated that they are a fine-tuner for the function of SGAs.

We further found that Met samples had equal similarities to DCIS and IDC in terms of their shared perturbed signaling pathways and driver genes. However, Met had more unique disturbed pathways compared to DCIS and IDC. This probably reflects a higher chance of Met samples resulting from neutral evolution, as this has been shown to be more prominent in the absence of selection pressure [18,30]. Therefore, we speculated an early divergence of metastases at the DCIS time-point which we corroborated in our phylogenetic analyses. In the phylogenetic analyses, the driver gene selection relied on the perturbation of pathways (SPIA) in the common gene list that originated from DCIS. Thus, DCIS samples served as a compass to define driver genes for the phylogenetic analyses. On the other hand, the correlation of DCIS samples to a specific subtype of breast cancer was very difficult. This was due to the fact that the expression of molecular subtyping markers shuffles during DCIS transition to IDC [33]. For example, HER2 overexpression is more frequent in DCIS compare to invasive cancer [34]. Such discordance applies to the hormone receptors as well [35]. Thus, we had only one type of DCIS that could not be correlated to other subtypes of breast cancer. Therefore, we were limited to the pooling of all IDC subtypes in our phylogenetic analyses.

Altogether, these findings were in line with recent studies that found that metastatic dissemination of breast and colon cancer cells is higher in the earlier stages of tumor progression compared to the advanced tumors, and that these cancer cells disseminated early on are the precursors of a high proportion of metastases [10,11,36]. In addition, recent evidence has indicated the uniformity of driver gene alterations, even at the single nucleotide level, in primary tumor samples, and in metastases of breast as well as pancreatic cancers [37,38,39], suggesting an early divergence of metastases from the primary tumor. Moreover, spatial analyses of invasive foci at the DCIS sites showed that genomic evolution occurred prior to the invasion through a multiclonal invasion model [40].

## 4. Materials and Methods

### 4.1. Data Collection, Preprocessing, and Identification of Differentially Expressed Genes

Gene expression microarray raw data were retrieved from the Gene Expression Omnibus (GEO) database (see GEO accession numbers in Figure S1a). We then discarded low-quality samples (102 samples were excluded) to focus on changes that triggered transitions between normal and DCIS, and DCIS and IDC or metastasis without extraneous interferences. All samples with no clear clinical annotation were also removed. Afterwards, Affy batch objects were created by the affy package in R [41] and the GCRMA package [42] to be used for preprocessing the datasets (including background correction, normalization, log transformation, and quality control). The identification of differentially expressed genes (DEG) was done in four states: normal vs. DCIS, normal vs. IDC, DCIS vs. IDC, and primary vs. metastasis via limma [43] through NetworkAnalyst (https://www.networkanalyst.ca/faces/home.xhtml) [44]. The combined *p*-values for the DEGs’ identification were obtained by using the state-combined Fisher’s method (adjusted *p*-values for DEGs from each study combined by Fisher’s method). DEGs within the mentioned states were named DEG1, DEG2, DEG3, and DEG4 for normal vs. DCIS, normal vs. IDC, DCIS vs. IDC states, and primary vs. metastasis, respectively (see all DEGs in Table S1). In order to evaluate the results obtained by microarray technology with RNA-seq data, the DEG2 signature was compared with the analogous DEG list from the largest available RNA-sequencing dataset in breast cancer and we calculated the significance via exact hypergeometric probability [26].

### 4.2. (Un)Supervised Clustering and Machine Learning

In an independent analysis, all samples obtained from the GPL96 (HG-U133A) and GPL570 (HG-U133_Plus_2) platforms were preprocessed with the FRMA package [45] and merged via the ComBatmethod [46] to remove their batch-to-batch variations and minimize the study bias effect. The reason why the FRMA preprocessing and ComBat methods were used for the unsupervised clustering section is that FRMA is a cutting-edge method for preprocessing when gene expression studies are supposed to be integrated directly. ComBat was also used to remove the batch effect between studies and to constitute the metadataset to perform a reliable visual inspection of the relationship between the phenotypes. The hierarchical clustering of the 60 most reproducible genes (genes with the highest log fold-change values and *q*-value < 0.01) in the metadataset (which was created after merging the individual GSE datasets) was done by the pairwise average-linkage method [47]. Furthermore, PCA was performed in order to identify the correlations between biologically distinct samples. In the supervised class prediction section, all GCRMA-normalized data were merged by ComBat and fed into the machine-learning (ML) models. In this study, four different supervised ML approaches for class prediction were used to properly assign samples to DCIS and IDC classes. The first ML tool was “Gradient Boosting Machine” (GBM) which produces a prediction model using decision trees in the form of an ensemble of weak predictions. In the GBM, a so-called regularization procedure named shrinkage controls the rate at which the loss of function is minimized. Smaller values of shrinkage result in greater accuracy, with the reason being that with smaller steps, the optimization is more precise [48]. The second ML tool was “Random Forest” which is an ensemble-learning-based method. This method works by constructing a multitude of decision trees at training time and outputting the class that is the mode of the classes or mean prediction (regression) of the individual trees [49]. The third ML tool was “k-nearest-neighbor algorithm” (KNN), which classifies a sample by assigning it to the label that is most frequently represented among the k-nearest samples [50]. The fourth ML tool was the “WeightedVotingXValidation” algorithm, which makes a weighted linear combination of relevant “marker” or “informative” genes obtained in the training set. This tool provides a classification scheme for new samples based on the leave-one-out cross-validation mode by iteratively leaving one sample out, training a model on the remaining data, and testing on the left-out samples [51]. All models obtained high-resolution discriminative features and were optimized by the minimum number of genes and maximum receiver operating characteristic area under the curve (ROC AUC). Finally, we developed a pipeline to iteratively evaluate the discriminative genes extracted from each ML model. We excluded top-ranked predicted genes and reran models for a maximum of nine rounds to prioritize each phenotype-specific feature and to test their correlation with the survival of patients.

### 4.3. Survival Analysis

The prognostic performance of gene signatures was assessed via the Cox model in terms of overall survival (OS) and recurrence-free survival analysis (RFS) using the Survexpress dataset [52], which included 1574 samples. The survival analysis was applied to highlight whether discriminative genes in each round of ML models had a correlation between the top-ranked genes obtained by ML models, and to optimize the sample separation of DCIS and IDC. We assumed that top-ranked discriminative genes between these two phenotypes (DCIS vs. IDC) must have a higher hazard ratio and correlation according to the concordance index (an index that correlates with the reliability of the created Cox model [53]). Furthermore, a survival analysis in terms of survival months was done using breast invasive carcinoma TCGA datasets, including 502 invasive samples for the detected genes involved in backward evolution. In all survival analyses, the “maximize risk groups” option was checked in order to optimize the risk group splitting using the Survexpress algorithm.

### 4.4. Integration of Copy-Number Alteration and Single Gene Alteration

Copy-number alteration (CNA) data were obtained from the Progenetix database (https://progenetix.org/, version 2017), including 76 DCIS and 1736 IDC samples (Table S2). A prototype map of CNAs was generated for chromosomal regions that had more than 10% gain or loss of alteration. Progressive gain and loss prototypes were defined for regions that already had a gain or loss in DCIS samples and further increased gain or loss in IDC samples. Regressive gain and loss prototypes were defined for regions that already had a gain or loss in DCIS samples and experienced a further gain or loss in IDC samples. Subsequently, the DEG3 gene list was cross-sectioned with DEG1 and DEG2 to generate a common and a disparate gene list. DEG3 included 1098 genes divided into 508 common and 590 disparate gene lists. Common lists were those genes shared between three DEGs, and disparate lists were genes shared between DEG2 and DEG3. Moreover, these common and disparate DEG lists were divided into common within CNA (CWC) regions, common outside of CNA (COC) regions, disparate within CNA (DWC) regions, and disparate outside of CNA (DOC) regions (Table S1). For the correlation analyses between single gene alterations (SGAs) and CNAs, we calculated the density of SGAs per megabase pair (density of SGA/Mbp) for each CNA prototype (Table S3).

### 4.5. Network Analysis

Reconstruction, visualization, and statistical analyses of the protein‒protein interaction networks (PPINs) were done using NetworkAnalyst [44] and STRING [53]. PPINs were created based on molecular interactions from multiple biological interaction databases including innateDB [54], IntAct [55], MINT [56], DIP [57], BIND [58], and BioGRID [59]. We reconstructed specific PPINs for the COC, CWC, DOC, and DWC regions. Network-based pathway-enrichment analysis was performed using the Reactome, KEGG, and GO modules through NetworkAnalyst. All *p*-values in this section were calculated at the default setting of NetworkAnalyst and STRING.

### 4.6. Enrichment Analysis

Gene-enrichment analysis of PPINs was done using the Reactome database through NetworkAnalyst. Only significantly enriched pathways (corrected *p*-value < 0.05) were considered. Moreover, signaling pathway impact analysis (SPIA) was applied to assess unusual signaling perturbations in pathways related to common or all (common + disparate) gene sets, and to analyze the fine-tuning functionality effect of backward evolution-related genes. According to the SPIA results, KEGG pathway-related genes were significantly perturbed in at least one of the common, all, and specific DCIS-related pathways (Table S7), as retrieved from the Molecular Signatures Database (MSigDB, http://software.broadinstitute.org/gsea/msigdb/index.jsp).

### 4.7. NGS Data and Point-Mutation Analysis

In order to find the mutation signatures related to the DCIS, IDC, and metastasis stages, we used COSMIC v89 (https://cancer.sanger.ac.uk/cosmic), which contained 2663 and 245 samples for IDC and DCIS, respectively. Moreover, 3607 IDC, 9 DCIS, and 86 metastatic samples were retrieved from cBioPortal (http://cbioportal.org; see [60,61]). Mutation signatures for each cancer stage were obtained from the aforementioned data by defining 5%, 2.5%, and 5% as the cutoff frequencies for DCIS, IDC, and metastasis, respectively. We assumed a lower cutoff for the IDC samples due to the higher heterogeneity in the population.

### 4.8. Identification of Driver Genes

We used the DawnRank method for the detection of driver genes. In principle, this method integrates PPINs with gene expression data and DNA-based somatic alterations, which enabled us to find the unique driver genes of which changes could deeply perturb the influential pathways [29,62]. On the other word, DawnRank is an elevated version of SPIA which adds genomic data to the analyses. We changed and adapted SPIA for our analyses. Here, we added the frequently mutated genes of each stage to the DEGs of that stage and ran PPIN analyses of the new gene list. Then we sorted all nodes with a degree ≥20 and ran KEGG pathway analyses to find the enriched pathways for highly connected genes. Finally, we did a comparison of our enriched pathways list with the perturbed pathways that characterized with the SPIA-detected driver genes of each stage.

### 4.9. Phylogenetic Analyses of Driver Genes

In order to study the evolutionary relationships between different stages of tumor progression, we used a mutation map of driver genes of DCIS, IDC, and Met, and a binary (0,1) dataset was created in which 1 represented genes with a point mutation in the driver gene lists and 0 represented nonmutated genes (Figure 6k). This binary matrix was analyzed by Parsimony analyses module in Past (v.2.17) software with its default setting.

### 4.10. Statistical Analysis

Statistical analyses and estimation of correlations in this study were performed using GraphPad Prism v.6. The significance was assessed via the Pearson correlation coefficient. The *p*-values reported for survival analysis were measured by the Cox regression hazard ratio and log rank tests. Paired and unpaired Student’s *t*-tests were performed when the data points passed a D’Agostino‒Pearson normality test. In cases where the variables were not distributed normally according to the D’Agostino‒Pearson test (*p* ≤ 0.05), we applied the Wilcoxon test. To compare numbers between different groups, we applied Fisher’s exact test, or if the samples numbered at least five in each condition of the χ^2^ tests. All *p*-values are two-tailed. All *p*-values and statistical tests are mentioned in either the figures or legends. To test the similarity of the gene expression data of microarray and RNA-seq (Figure S1b) data, we used the exact hypergeometric probability test from online tools (http://www.nemates.org/MA/progs/overlap_stats.html).

## 5. Conclusions

Altogether, this study suggested that the differences in the gene expression profiles of pre- and postinvasive breast cancer do not present a new foundation route of malignancy. Indeed, the fundamental genetic alterations of SGAs occurring in the earlier stages of tumor development and further SGAs in later stages serve, in a forward evolution model, as a fine-tuning mechanism of those primary changes. Remarkably, we were able to apply a backward evolution model for cancer genome evolution, in which CNAs are selective events and serve as means to reverse the expression/direction of a proportion of the earlier SGAs. Our methodology introduces a new pipeline for cancer evolution studies, and our results draw a global portrait of the evolution of malignancy in breast cancer.

## Supplementary Materials

The following are available online at https://www.mdpi.com/2072-6694/12/6/1460/s1, Figure S1: Samples and gene expression heatmaps, Figure S2: Prediction of the results of machine learning models and survival analyses, Figure S3: Correlation between number of genes and survival, Figure S4: Direction of changes in CNA and SGA, Figure S5: Forward and backward evolution and fine-tuning concepts, Figure S6: Common-Met and the heatmap of driver genes, Table S1: DEG lists, Table S2: Genomatix data, Table S3: CNA prototype map, Table S4: SGA density, Table S5: Networks and GSEA, Table S6: Homo/Hetero Nodes, Table S7: SPIA for DEG 1, 2, and 3, Table S8: Met-DEG, Table S9: SPIA for DEG 1, 2, and 4, Table S10: Driver genes.
