# Supplementary material for: Omics Integration Analyses Reveal the Early Evolution of Malignancy in Breast Cancer
Source: Cancers (Basel). 2020 Jun 4;12(6):1460. doi: 10.3390/cancers12061460 (PMC7352609; doi:10.3390/cancers12061460)
Supplement: Supplementary file 1 [file cancers-12-01460-s001.zip › cancers-786935-final check-supplementary materials/Supplementary Figures.docx]

Article

Omics Integration Analyses Reveal the Early Evolution of Malignancy in Breast Cancer

Shamim Sarhadi, Ali Salehzadeh-Yazdi, Mehdi Damaghi, Nosratollah Zarghami, Olaf Wolkenhauer and Hedayatollah Hosseini


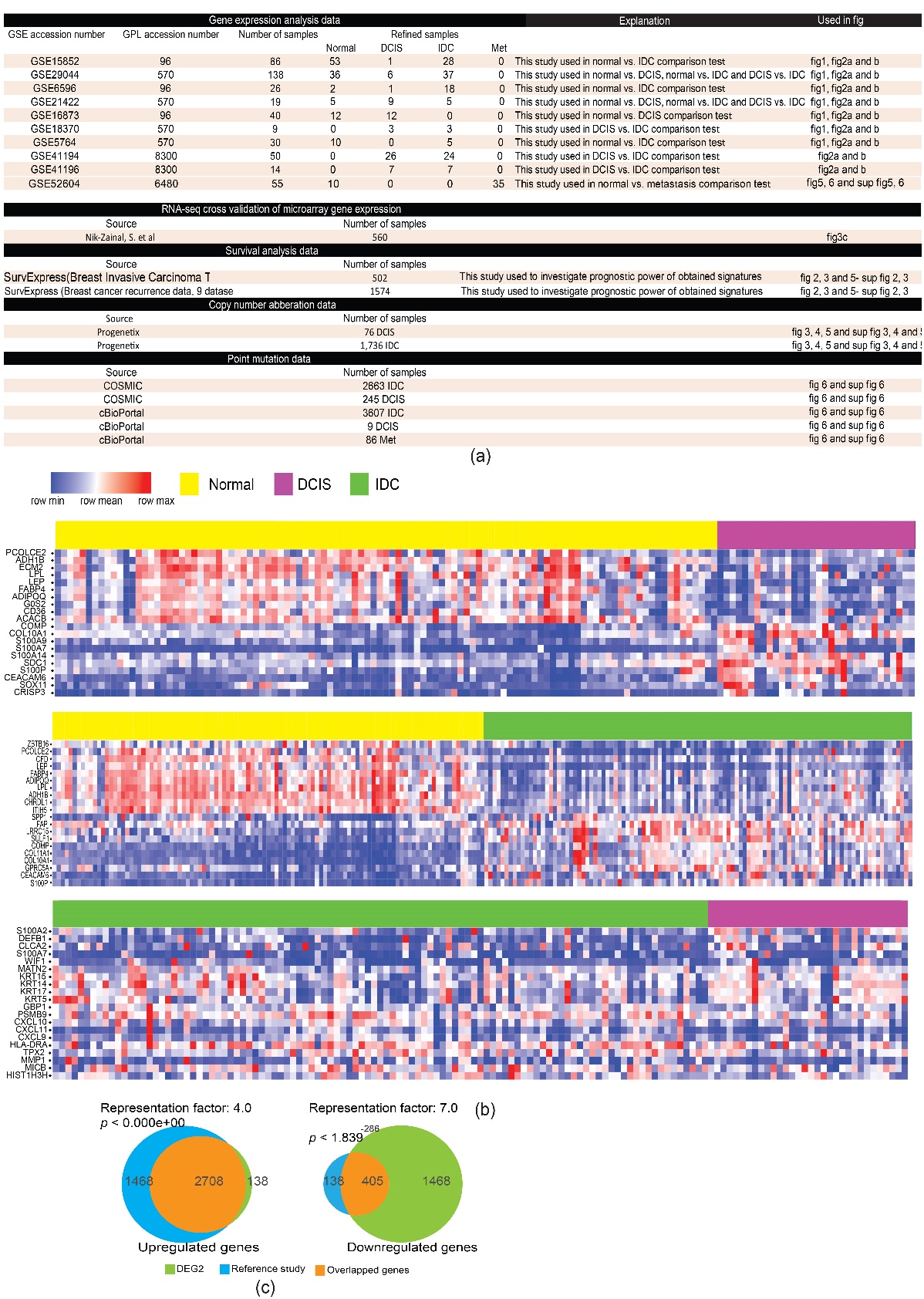


**Figure S1.** Samples and gene expression heatmaps. (**a**) List of samples, datasets and their characteristics used in this study. (**b**) Heatmap of 20 genes with the highest LFC in Normal, DCIS and IDC samples. Rows (genes) clustered by Pearson correlation method and average-linkage hierarchical clustering method and samples (columns) clustered by their phenotypes (Hierarchical clustering was not applied on columns). (**c**) Venn diagrams show a high overlap between DEG2 (IDC vs Normal) and analogous RNA-seq data [22]. Evaluation *p*-value was calculated using the exact hypergeometric probability test. The RF that presented the number of shared genes divided by the expected number of shared genes resulting from two independent groups. RF more than 1 shows more overlap than expected and vice versa.


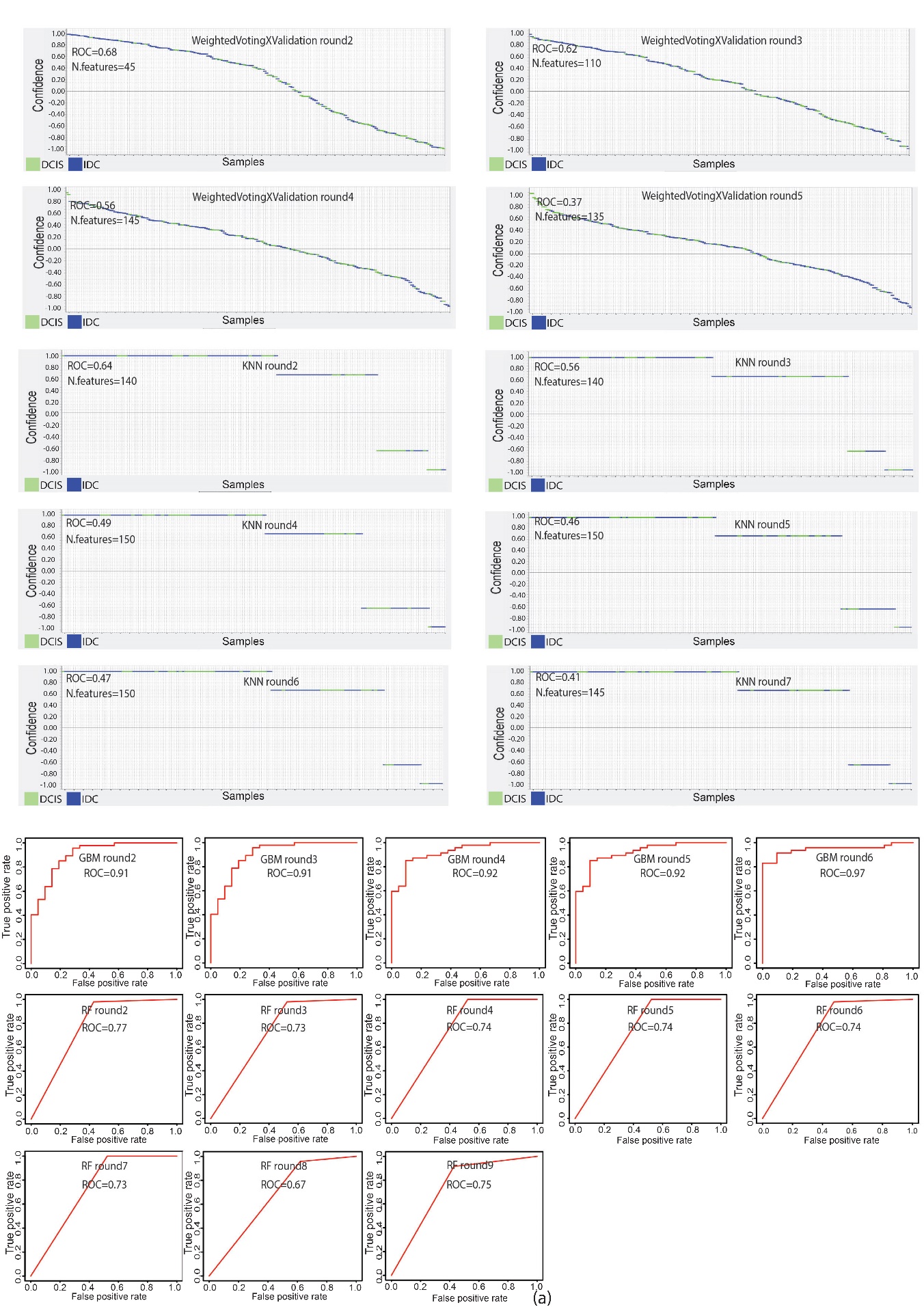


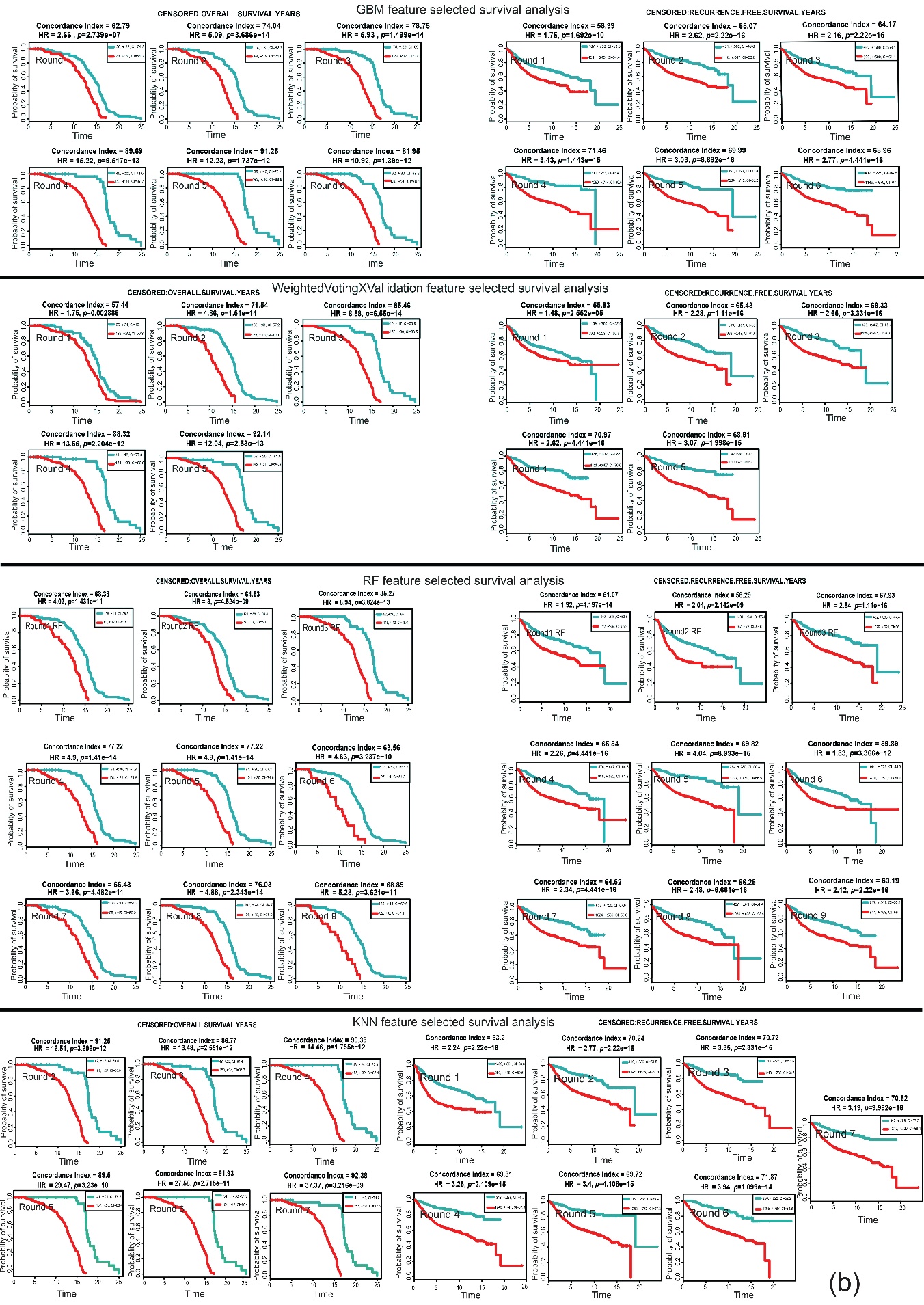

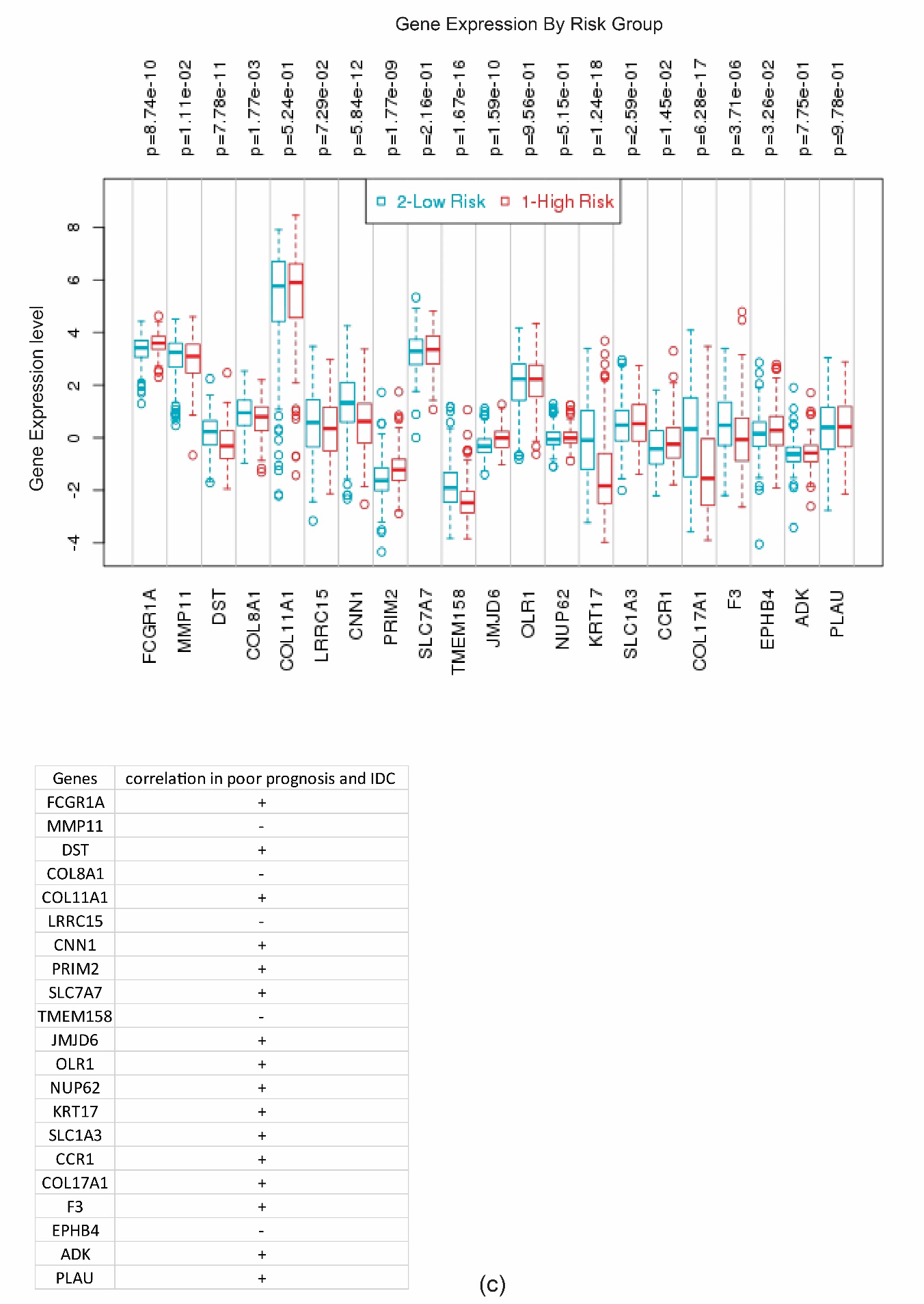


**Figure S2.** Prediction of the results of machine learning models and survival analyses. (**a**) Machine learning (ML) class prediction models that were optimized by four different approaches including GBM, RF, KNN and weightedVotingXValidation. Plots represent the specificity and sensitivity of models through ROC values for the features, which are shown in Fig 2b. (**b**) Kaplan-Meier survival analyses (OS and RFS) for gene features selected in each round of ML models presented in Figure 2b. (**c**) Example of correlation between gene expression in the poor (red) and good (blue) prognosis samples obtained for survival analyses. Table in the below of gene expression graph represents the concordance between expression of that given gene in poor prognosis samples and the real expression direction in the IDC samples.


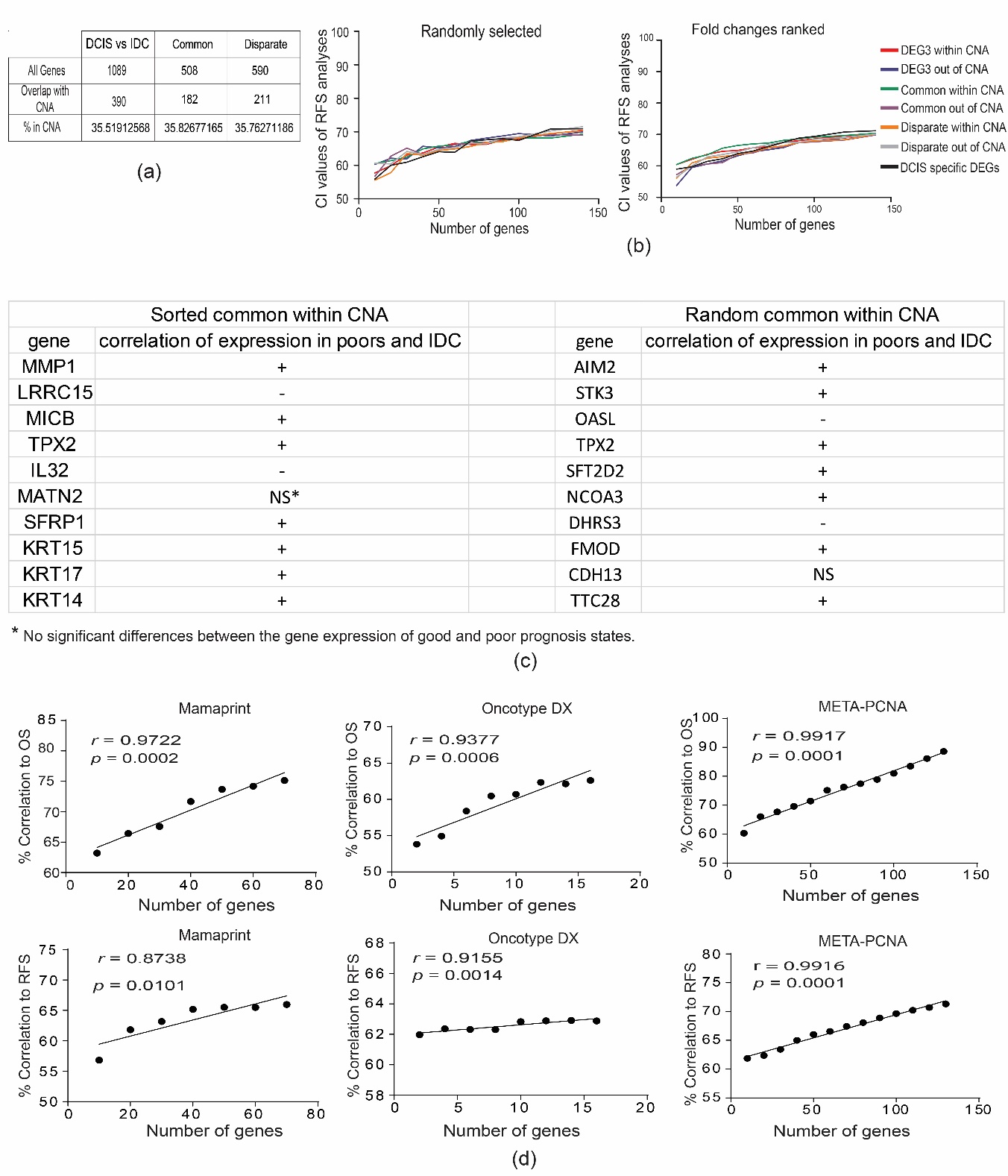


**Figure S3.** Correlation between number of genes and survival. (**a**) Number of common and disparate genes and their contribution in CNAs. (**b**) Correlation of concordance index (CI) obtained from relapse free survival (RFS) analyses with the number of genes selected randomly (left) or based on ranked fold changes (right). Genes are selected from common, disparate, DCIS specific and DEG 3 (IDC vs DCIS) within and out of CNV. (**c**) Two example of correlation between gene expression in the poor and good prognosis samples obtained for survival analyses in Figure 3d. Table represents the concordance between the expression of that given gene in poor prognosis samples and the real expression direction in the IDC samples. (**d**) Correlation of CI of overall survival and recurrence free survival CI with the number of genes from available gene signatures including Mamaprint, META-PCNA and Oncotype DX. Evaluation *p*-values in **d** was calculated using the Pearson correlation test.


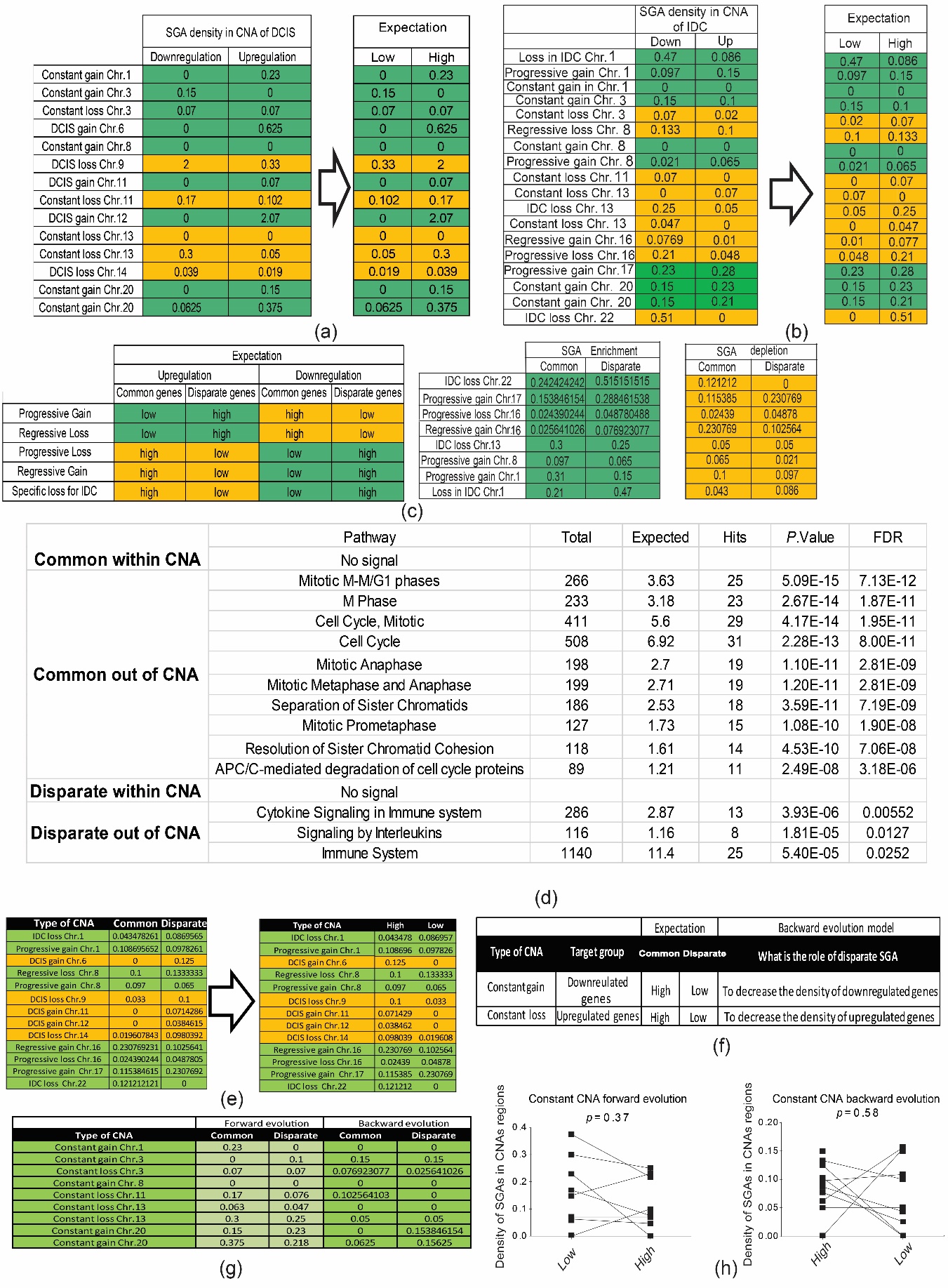


**Figure S4.** Direction of changes in CNA and SGA. **(a** and **b**) Scheme table of the density of SGAs (number of SGAs per Mb) in DCIS CNA (a) IDC CNA (b) prototypes show in the left table and the direction of our predictions show in the right table. The yellow squares show loss-CNAs where we expect the density of down-regulated genes would be high. Therefore, in the right table we flipped the numbers of yellow squares to bring “down” numbers in the “high” columns. The green squares present unchanged numbers between left and right columns. (**c**) Scheme tables present *forward evolution* model for relation of SGAs through DCIS (common SGAs) to IDC (disparate SGAs). Note that we evaluated all SGAs (common and disparate) in the IDC CNAs prototypes as the final evolved version of CNAs. The green color predicts the enrichment of SGAs and the yellow one predicts the depletion of SGAs. The right tables present real numbers of SGAs density. (**d**) Enrichment pathway analysis of common SGAs within CNAs (CWC), common out of CNAs (COC), disparate within CNAs (DWC) and disparate out of CNAs (DOC) networks by Reactome enriched functional subnetworks in the main PPIN. (**e**) This table presents the density of SGA numbers for CNA-prototypes with a possible *backward evolution* function that is mentioned in the table in Figure 4i. (**f**) Scheme table for the *backward evolution* concept in CNA constant prototypes. (**g**) SGAs number for *forward* and *backward* *evolution* models in the CNA constant prototypes (**h**) Evaluation of *backward* and *forward evolution* model in constant CNAs. Evaluation *p*-values in **h** was calculated using the Wilcoxon matched-pair signed rank test.


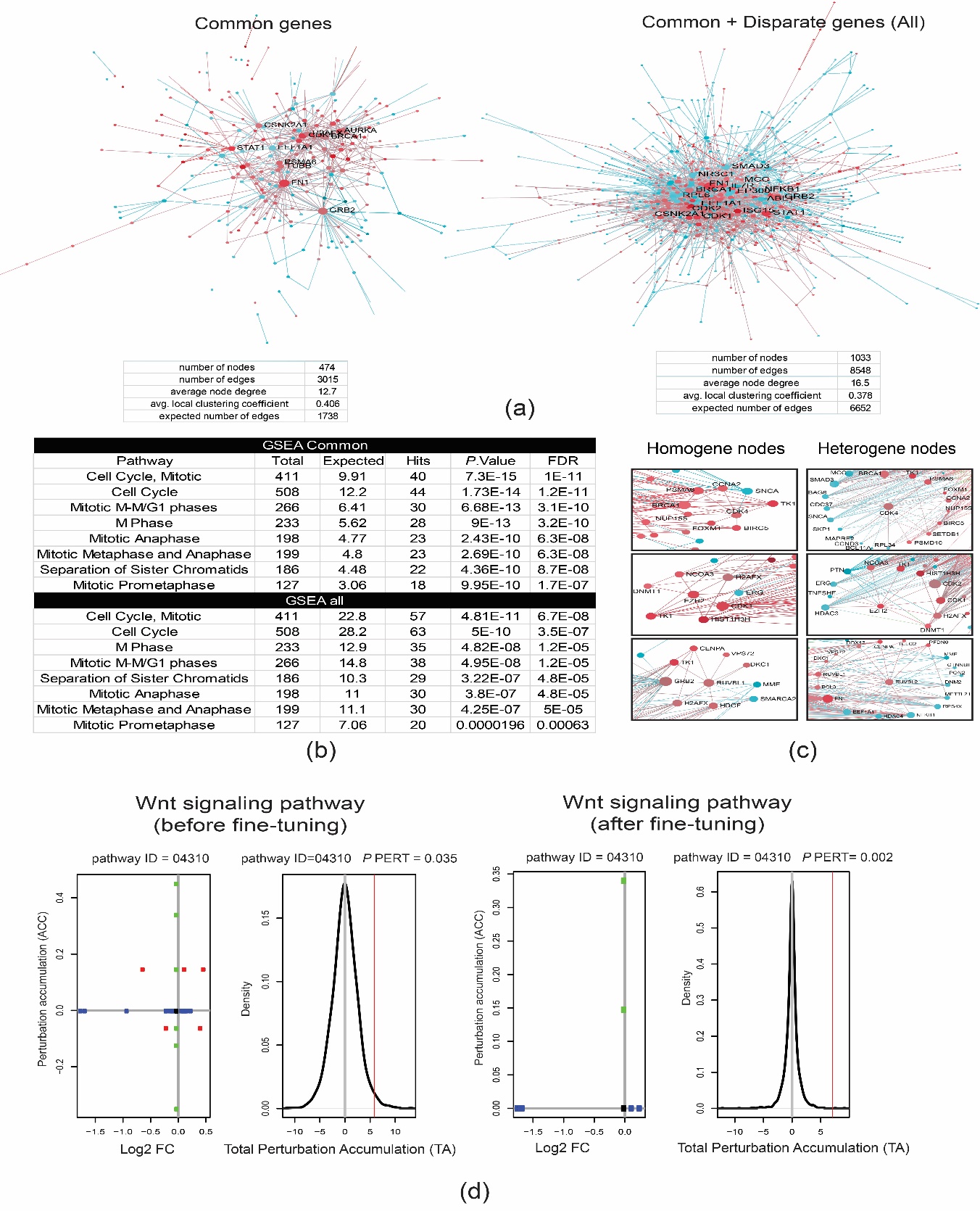


**Figure S5.** Forward and backward evolution and fine-tuning concepts. (**a**) PPINs and its related-statistics associated to common SGAs and all DEG3 (all common and disparate SGAs). PPINs were created by mapping gene sets to the Networkanalyst database. (**b**) Enrichment pathway analysis for common and all DEG3 networks (in panel a). Top 10 enriched pathways presented for each gene set profile. (**c**) Examples of homogene and heterogene nodes. (**d**) Examples of SPIA analyses for DEG3 before and after adding eight *backward evolution* genes. In this plot, the horizontal axis represents the *p*-value (minus log of) corresponding to the probability of obtaining at least the observed number of genes (NDE) in the given pathway randomly. The vertical axis represents the *p*-value (minus log of) corresponding to the probability of obtaining the observed total accumulation (tA) or more extreme on the given pathway randomly. Unchanged genes are assigned 0 log2 fold-change. The null distribution of the net accumulated perturbations is also given (right panel). The observed net accumulation tA with the real data is shown as a red vertical line (see Ref 28 in the main text).


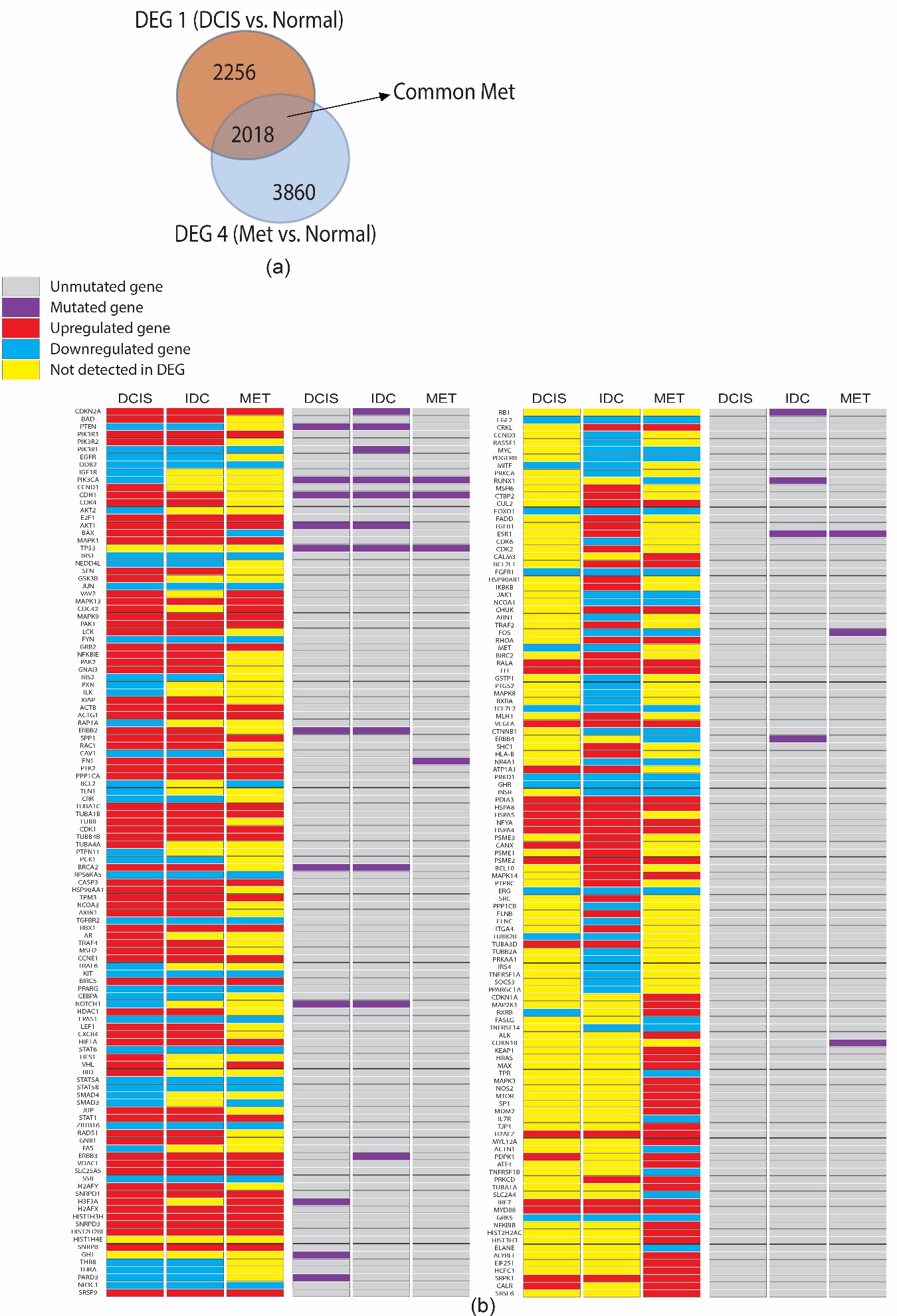


**Figure S6.** Common-Met and the heatmap of driver genes. (**a**) Graphic demonstrates common-Met gene expression profile (Table S8). (**b**) Heatmap of all driver genes in DCIS, IDC, and Met with the direction of expression and mutation state.

**Supplementary Tables:** please view at the excel files.

| 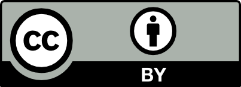 | © 2020 by the authors. Licensee MDPI, Basel, Switzerland. This article is an open access article distributed under the terms and conditions of the Creative Commons Attribution (CC BY) license (http://creativecommons.org/licenses/by/4.0/). |
| --- | --- |
